# The complete mitochondrial DNA sequence of Yimeng wool rabbit

**DOI:** 10.1080/23802359.2019.1687022

**Published:** 2019-11-11

**Authors:** Chang-Yu Yao, Yao-Yao Li, Ling-Xiao Liu, Chao Ma, Yun-Guo Liu, Ye-Hai Liu

**Affiliations:** a Department of Otorhinolaryngology Head and Neck Surgery, The First Affiliated Hospital of Anhui Medical University, Hefei, China;; b College of Life Sciences, Linyi University, Linyi, China;; c Marine College, Shandong University (Weihai), Weihai, China;; d Linyi Academy of Agricultural Sciences, Linyi, China

**Keywords:** Yimeng wool rabbit, *Oryctolagus cuniculus*, mitochondrial genome

## Abstract

Yimeng wool rabbit is a national breed of geographical indication in China. The complete mitochondrial genome sequence of Yimeng wool rabbit was first determined in this study (Accession number MN296708). The mitogenome (16,740 bp) consists of 22 tRNA genes, 2 ribosomal RNA genes, 13 protein-coding genes, and 1 control region (D-loop region). The complete mitochondrial genome sequence of the Yimeng wool rabbit enriches data resource for further study in genetic mechanism and classification.

Wool rabbit, also known as Angora rabbit, originated in Asia minor area (Liang 1985). Four main breeds of Angora rabbit exist: English Angora, French Angora, German Angora, and Chinese Angora (Zou 2012). Yimeng wool rabbit (*Oryctolagus cuniculus*) is a kind of special economic animal with broad market prospect, its wool has high economic value. China has many excellent native breeds of rabbit, including Yimeng wool rabbit in Shandong Province, which has unique characteristics of higher wool production and better wool quality (Zhang 2018). In this study, the complete mitochondrial genome of Yimeng wool rabbit was sequenced and characterized in detail. Sample was collected from Mengyin City (35°43′N, 117°57′E), Shandong Province, China, in July 2019. The specimen of Yimeng wool rabbit, named as YimengWR-01, was stored in College of Life Sciences, Linyi University, Linyi, China. The total genomic DNA was extracted from Yimeng wool rabbit muscle according to Liu et al. (2012, 2014). The complete mitochondrial genome was sequenced using a shotgun approach and assembly. DNA sequence was analyzed using MEGA 7 (Kumar et al. 2016). Protein-coding genes were analyzed by ORF Finder (http://www.ncbi.nlm.nih.gov/gorf/gorf.html) using the invertebrate mitochondrial code. The tRNA genes were identified by ARWEN (Laslett and Canback 2008) and tRNA-scan SE (Lowe and Eddy 1997).

The complete mitochondrial genome of Yimeng wool rabbit (Accession number MN296708) was 16,740 nucleotides long, of which 15,383 nucleotides are coding DNA and 1357 nucleotides are non-coding DNA. It consists of 22 tRNA genes, 2 ribosomal RNA genes, 13 protein-coding genes, and 1 control region (D-loop region). The structure and composition of the mitochondrial genome is also comparable to the case of other vertebrates (Gissi et al. 1998; Xu et al. 2015; Liu et al. 2016; Shan and Liu 2016; Li, Liu, Sui et al. 2019; Li, Liu, Zhang et al. 2019). A phylogenetic tree was constructed based on the comparison of the complete mitochondrial genome sequences with other Leporidae species using the neighbor-joining method (Figure 1). There are 21 overlapping regions (total 111 bp) and 11 intergenic spacers (total 63 bp) among the genes. The total base composition of the mitochondrial genome is 31.47% A, 28.25% T, 26.61% C and 13.67% G, and an A + T (59.72%)-rich feature occurs in the Yimeng wool rabbit. To investigate the nucleotide bias, skew for a given strand was calculated as (A - T)/(A + T) or (G - C)/(G + C) (Perna and Kocher 1995). The AT and GC skews for the Yimeng wool rabbit mitochondrial genome were 0.054 and −0.321, respectively; this finding indicated that the strand that encoded genes contained more A and C than T and G, and this skew was evidence of codon usage bias. The total length of 13 protein-coding genes is 11,427 bp. Except *ND2* and *ND5* protein-coding genes using the start codon ATT, *ND3* using the start codon ATA, other ten protein-coding genes use the start codon ATG. When it comes to stop codons, *ND2* and *ND4L* terminate with TAA, whereas *COX2* and *ND5* terminate with TAG, *ND6* terminate with AGG, respectively. In addition, eight genes terminate with incomplete stop codon T–– that is the 5′ terminal of the adjacent gene, which presumptively formed a complete stop codon by post-transcriptional polyadenylation (Anderson et al. 1981). All the mitogenome genes were encoded on the H strand except for *ND6* and eight tRNA genes (tRNA-Gln, Ala, Asn, Cys, Tyr, Ser, Glu, Pro). The *12S rRNA* (958 bp) gene and *16S rRNA* (1582 bp) gene were located between the *tRNA*-*Phe* and *tRNA*-*Leu* genes and separated by the *tRNA*-*Val* gene, and the result was the same as the other mammals. The lengths of 22 tRNA genes range from 60 to 74 bp. The D-loop region locates between *tRNA*-*Pro* and *tRNA*-*Phe* with a length of 1294 bp. The small non-coding region, a putative origin of the light-strand replication, was located between *tRNA*-*Asn* and *tRNA*-*Cys* genes in the length of 31 bp. The data would facilitate further investigations of phylogenetic relationships within Leporidae.

**Figure 1.**
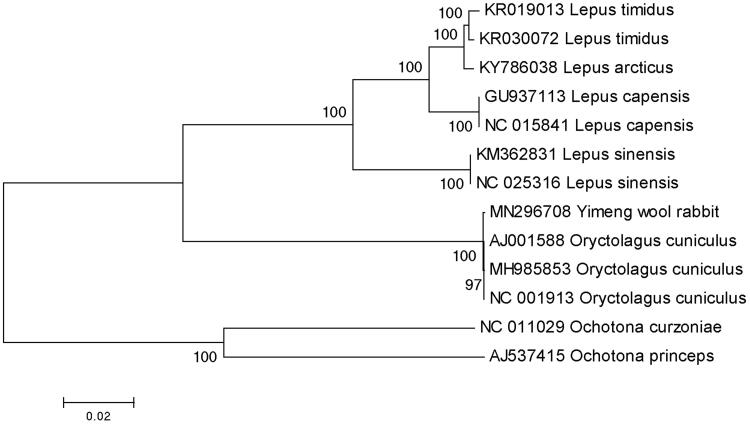
A phylogenetic tree constructed based on the comparison of complete mitochondrial genome sequences of the Yimeng wool rabbit (*Oryctolagus cuniculus*) and other five species of Leporidae family. They are *Oryctolagus cuniculus* (European rabbit), *Lepus capensis* (cape hare), *L. sinensis* (Chinese hare), *L. timidus* (moutain hare), *L. arcticus* (arctic hare). *Ochotona princeps* and *Ochotona curzoniae* are using as an outgroup. Genbank accession numbers for all sequences are listed in the figure. The numbers at the nodes are bootstrap percent probability values based on 1000 replications.
